# Intensification of upfront chemotherapy for patients with myeloid blast phase CML: a single center experience

**DOI:** 10.1007/s00277-024-06045-8

**Published:** 2024-10-23

**Authors:** Benjamin J. Lee, Shawn P. Griffin, Jean Doh, Stefan O. Ciurea, Deepa Jeyakumar, Piyanuch Kongtim, Kiran Naqvi

**Affiliations:** 1 Department of Pharmacy, Chao Family Comprehensive Cancer Center, University of California Irvine Health, 101 The City Drive South, Bldg. 23, Rm 275, Orange, CA 92868 USA; 2 Department of Clinical Pharmacy Practice, School of Pharmacy & Pharmaceutical Sciences, University of California, Irvine, CA USA; 3 Department of Medicine, Division of Hematology Oncology, Chao Family Comprehensive Cancer Center, University of California Irvine Health, Orange, CA USA

**Keywords:** Chronic myeloid leukemia, Blast-phase CML, Tyrosine kinase inhibitor, Myeloid blast crisis, “7 + 3” chemotherapy

## Abstract

Outcomes for patients with myeloid blast phase chronic myeloid leukemia (CML-MBP) are dismal, and no preferred chemotherapy regimen has been identified. Recent studies have suggested a higher response rate with administration of timed-sequenced regimens (TSR) (purine analog, high-dose cytarabine, anthracycline) in high-risk acute myeloid leukemia patients. We retrospectively evaluated outcomes of newly diagnosed CML-MBP patients consecutively treated at our institution with a TSR or standard-dose cytarabine and an anthracycline (“7 + 3”) combined with a tyrosine-kinase inhibitor (TKI) between 2011 and 2023. Endpoints of interest included hematologic response, clinically significant cytogenetic response (CSCR) defined as achieving at least a minor cytogenetic response (Ph + metaphases 0%-≤65%) after induction therapy, event-free survival (EFS), and overall survival (OS). A total of 18 patients with CML-MBP were included of whom 9 (50%) received a TSR and 9 (50%) received “7 + 3”. Hematologic response (55.6% vs. 55.6%) and CSCR (25% vs. 37.5%) were similar between TSR- and “7 + 3” treated patients. Twelve patients (66.7%) experienced at least one grade ≥ 3 non-hematologic, end-organ toxicity with 33.3% and 11.1% of TSR- and 7 + 3-treated patients, respectively, experiencing at least two. Our data suggests that intensification of upfront chemotherapy does not appear to improve treatment outcomes in CML-MBP patients however, further studies are warranted to confirm these findings involving a larger cohort.

## Introduction

Chronic myeloid leukemia (CML) is a myeloproliferative neoplasm uniquely characterized by a sole reciprocal translocation between chromosomes 9 and 22, producing the BCR-ABL1 oncogenic fusion or Philadelphia chromosome [Ph(+)]. Despite the advent of BCR-ABL targeting tyrosine kinase inhibitors (TKIs), a small percentage of patients progress from a prior chronic or accelerated phase (CP/AP) to blast phase CML (CML-BP). In addition, patients may present with *de novo* CML-BP. Although myeloid blast phase CML (CML-MBP) has the hematologic characteristics of acute myeloid leukemia (AML), it is marked by therapy resistance and dismal treatment outcomes. The two-drug intensive chemotherapy regimen, “7 + 3,” consisting of standard-dose cytarabine in combination with daunorubicin or idarubicin, is a widely established standard of care in the treatment of newly diagnosed, non-M3 AML patients [1]. Several studies have evaluated this chemotherapy backbone plus a TKI for patients with CML-MBP with encouraging results [2, 3]. Recent studies have described positive outcomes with a three-drug, timed-sequenced regimen (TSR) consisting of a purine analog followed by high-dose cytarabine and an anthracycline in the upfront setting for AML and could potentially improve outcomes for CML-MBP patients [4–6]. However, chemotherapy alone failed to produce durable responses in these patients. In a large retrospective study of CML-MBP patients, combination therapy with a TKI led to significantly higher response rates and longer survival compared to TKI or chemotherapy alone [7]. In order to determine if a three-drug TSR in combination with a TKI leads to improved outcomes over “7 + 3” regimen with a TKI, we conducted a retrospective single-center study of front-line CML-MBP patients.

## Methods

We reviewed all adult (≥ 18-years old) CML-MBP patients consecutively treated with chemotherapy and a TKI at our institution between January 2011 and August 2023. Patients were assessed for eligibility if they had a diagnosis of CML based on the presence of translocation (9;22) and exhibited ≥ 20% myeloblasts in the bone marrow, with or without extramedullary involvement. Patients were excluded if they had received any other prior therapy for CML-MBP except for hydroxyurea. Patients with mixed myeloid/lymphoid phenotype and Ph(+) acute leukemia were also excluded. This study was approved by the University of California Irvine Institutional Review Board and a waiver of informed consent was obtained.

Outcomes of interest included hematologic response, clinically significant cytogenetic response (CSCR), event-free survival (EFS), and overall survival (OS). Hematologic response was defined as a composite of complete remission (CR) and CR with incomplete hematologic recovery (CRi) [8], whereas CSCR was defined as achieving at least a minor cytogenetic response (Ph + metaphases 0%-≤65%) after induction therapy. The presence of 0-35% and 36-65% of metaphases exhibiting the Ph(+) chromosome were further classified as major and minor cytogenetic responses, respectively. EFS was defined as the time from CML-MBP treatment initiation to the time when primary refractory disease was confirmed (i.e., when failure to achieve a response to induction was determined), relapse after initial hematologic response, or death from any cause. OS was defined as the time from treatment initiation to death from any cause. Hematologic and cytogenetic responses were assessed at hematologic recovery or by day 35 of induction treatment, whichever occurred first. Major molecular response (MMR) or MR^3^ was defined as a BCR-ABL1/ABL1 ratio ≤ 0.1% on the international scale (IS). MR^4^ and MR^4.5^ were defined as a BCR-ABL1/ABL1 ratio ≤ 0.01% and ≤ 0.0032% on the IS, respectively. Lastly, adverse events following induction chemotherapy were assessed for all patients and severe end-organ dysfunction was defined as grade ≥ 3 toxicity in alignment with the National Cancer Institute Common Terminology Criteria for Adverse Events (CTCAE; version 5.0). Multi-organ toxicity was defined as impairment of at least two of the following systems: lungs, cardiovascular system, gastrointestinal tract, kidneys, and liver. Patients were censored at their last follow-up if they were alive without the event of interest. Continuous variables were evaluated using Wilcoxon rank sum or Student’s t-test, while categorical variables were evaluated with the chi-square or Fisher’s exact test, as appropriate. Time-to-event endpoints were summarized via the Kaplan-Meier method, and treatment groups were compared using a 2-sided log-rank test. The impact of response to induction therapy and allogeneic hematopoietic stem cell transplantation (aHSCT) on OS was assessed utilizing landmark analysis whereby patients alive at 1- and 4-months, respectively, and achieving hematologic response in the latter, were included. Statistical analyses were performed using Intercooled Stata, version 18.0 (StataCorp, College Station, TX).

## Results

### Baseline characteristics

A total of 18 patients with CML-MBP were included of whom 9 (50%) received a TSR and 9 (50%) received “7 + 3”. The median [IQR] age was 51 [39–67] years and 61.1% were male. Overall, 13 patients (72.2%) progressed from a prior CP/AP of whom 84.6% (*n* = 11) received at least one prior TKI before MBP transformation (Table 1). The remaining two patients were managed with hydroxyurea and leukapheresis at outside hospitals for their CML-CP and were lost to follow-up before presenting to our institution with CML-MBP. Poor-genetic risk characteristics were present in 13 patients (72.2%), of whom 7 (38.9%) had complex cytogenetics; 1 (5.6%), 2 (11.1%), 6 (33.3%), 4 (22.2%), and 1 (5.6%) had a TP53, ASXL1, EVI1-MECOM, RUNX1, and DEK-NUP214 mutation, respectively. BCR-ABL resistance testing was performed in all included patients; three acquired the T315I point mutation and received ponatinib with induction therapy, while one patient in the “7 + 3” cohort had a F317L mutation, and one in the TSR cohort a E225K mutation. The median ECOG at treatment initiation was 1 (range, 0–3) with 33.3% of patients with a performance score ≥ 2.

### Hematologic, cytogenetic, and molecular response

Hematologic response after cycle 1 induction chemotherapy was achieved in 55.6% (*n* = 10) of all patients, 5 (55.6%) patients in the “7 + 3” and TSR cohorts each. Further details outlining responses are available in Tables 1 and 2. Patients with lower (< 50% vs. ≥ 50%) bone marrow blasts (66.7% vs. 33.3%) at diagnosis and *de novo* CML-MBP (80% vs. 46.2%) were more likely to achieve hematologic response whereas complex cytogenetics (57.1% vs. 54.6%) or poor genetic risk (61.5% vs. 40%) did not seem to affect response. Among those who progressed from a previous CP/AP CML to CML-MBP, response was comparable between TSR and “7 + 3”-treated patients (42.9% vs. 50%; *P* > 0.99). Receipt of third-generation TKI, ponatinib, appeared to be associated with improved hematologic response yet did not reach statistical significance both in the full cohort (66.7% vs. 50%, *P* = 0.64) and for those with prior CP/AP (66.7% vs. 28.6%; *P* = 0.29). The combination of TSR and ponatinib (*n* = 4) did not produce higher hematologic responses when compared with all other therapeutic combinations (50% vs. 57.1%). Among patients with poor-genetic risk characteristics, receipt of a TSR did not affect hematologic response outcomes (57.1% vs. 66.7%; *P* > 0.99). Cytogenetic response was not available in two patients who experienced early mortality (< 30 days from treatment initiation), both with refractory disease. CSCR was seen in 25% (*n* = 2/8) and 50% (*n* = 4/8) of evaluable TSR and “7 + 3”-treated patients, respectively. Patients who received ponatinib did not achieve a higher rate of CSCR compared to those who received dasatinib (33.3% vs. 40%; *P* > 0.99). Of the 10 patients who achieved hematologic response post-induction, five (50%) achieved at least a MMR with a median time of 3.3 months. Four patients relapsed with a median [IQR] onset of 2.9 [2.5–4.9] months. Seven patients in total (3 relapse and 4 refractory) received salvage therapy of whom only two (28.6%) achieved a hematologic response, one with mitoxantrone, etoposide, and cytarabine and another with a TSR (Table 2). In total, five patients (27.8%) proceeded to aHSCT.

**Table 1:** Patient baseline characteristics and outcomes

Variables ^a^	All Patients (*n* = 18)	TSR ^b^ (*n* = 9)	7 + 3 ^c^ (*n* = 9)
**Baseline Characteristics**			
Male sex, n (%)	11 (61.1)	4 (44.4)	7 (77.8)
Age (years)	51 [39–67]	53 [39–67]	46 [38–52]
ECOG PS	1 (0–3)	1 (1–3)	1 (0–2)
0–1	12 (66.7)	5 (55.6)	7 (77.8)
2–3	6 (33.3)	4 (44.4)	2 (22.2)
WBC count (cells x 10^3^/uL)	95.8 [42.4–280]	125.9 [42.4–250]	85.7 [48.1–331]
Bone marrow blasts (%)	40 [30–80]	40 [30–40]	40 [30–80]
Blasts ≥ 50%, n (%)	6 (33.3)	2 (22.2)	4 (44.4)
De Novo CML-MBP, n (%)	5 (27.8)	3 (33.3)	2 (22.2)
Poor Genetic Risk,^d^ n (%)	13 (72.2)	7 (77.8)	6 (66.7)
Complex cytogenetics	7 (38.9)	3 (33.3)	4 (44.4)
TP53	1 (5.6)	1 (11.1)	0 (0)
ASXL1	2 (11.1)	1 (11.1)	1 (11.1)
EVI1/MECOM	6 (33.3)	4 (44.4)	2 (22.2)
RUNX1	4 (22.2)	3 (33.3)	1 (11.1)
DEK-NUP	1 (5.6)	0 (0)	1 (11.1)
Prior TKI exposure, n (%)	11 (61.1)	6 (66.7)	5 (55.6)
Induction TKI therapy, n (%)			
Dasatinib	12 (66.7)	5 (55.6)	7 (77.8)
Ponatinib	6 (33.3)	4 (44.4)	2 (22.2)
**Outcomes**			
Hematologic response, n (%)	10 (55.6)	5 (55.6)	5 (55.6)
CR	5 (50)	3 (60)	2 (40)
CRi	5 (50)	2 (40)	3 (60)
Cytogenetic response, ^e^ n/N (%)			
CSCR ^f^	6/16 (37.5)	2/8 (25)	4/8 (50)
Minor response ^g^	4/6 (66.7)	0/2 (0)	4/4 (100)
Major response ^g^	2/6 (33.3)	2/2 (100)	0/4 (0)
Best Molecular response, ^h, i^ n/N (%)	5/10 (50)	2/5 (40)	3/5 (60)
MMR (MR^3^)	2/10 (20)	1/5 (20)	1/5 (20)
MR^4^	0	0	0
MR^4.5^	3/10 (30)	1/5 (20)	2/5 (40)
Time to initial MR (months)	3.3 [2.8–4.6]	3.7 [2.8–4.6]	3.3 [3.0-5.8]
Time to best MR (months)	4.5 [3.0-4.6]	3.7 [2.8–4.6]	4.5 [3.0-8.8]
Proceeded to aHSCT, n (%)	4 (22.2)	2 (22.2)	2 (22.2)
EFS (months)	2.5 [0.5–9.6]	2.5 [0.9–3.4]	2.7 [0.5–20.7]
OS (months)	5.0 [2.1–20.7]	3.9 [2.1–19.6]	11.1 [2.7–20.7]
Relapse after response, n/N (%)	4/10 (40)	2/5 (40)	2/5 (40)
1-year EFS, %	13.3%	11.1%	16.7%
1-year OS, %	38.2%	31.8%	44.4%

*Abbreviations* aHSCT, allogeneic hematopoietic stem cell transplantation; CML, chronic myeloid leukemia; CR, complete remission; CRi, complete remission with incomplete hematologic recovery; ECOG, Eastern Cooperative Oncology Group; EFS, event-free survival; MBP, myeloid blast phase; NR, not reached; OS, overall survival; PS, performance status; TKI, tyrosine kinase inhibitor; TSR, time-sequenced regimen; WBC, white blood cell

^a^ All variables reported as median [IQR] unless otherwise stated

^b^ Time-sequenced regimens consisting of: fludarabine plus high-dose cytarabine and idarubicin (FIA or FLAG-Ida), cladribine plus high-dose cytarabine and idarubicin (CLIA), and cladribine plus high-dose cytarabine and mitoxantrone (CLAG-M)

^c^ Intensive chemotherapy consisting of 7 days of standard-dose cytarabine and 3 days of idarubicin or daunorubicin

^d^ Patients with one or more cytogenetic or molecular characteristics associated with poor outcomes

^e^ Cytogenetic response assessment not available in 2 treated patients who experienced early mortality within 30 days and with residual blasts

^f^ Defined as achieving at least a minor cytogenetic response (≤ 65%) after induction therapy

^g^ Minor and major response defined by presence of 0-35% and 36-65% of metaphases exhibiting the Ph(+) chromosome, respectively

^h^ MMR(MR^3^): BCR-ABL1/ABL1 ratio of ≤ 0.1% on the international scale (IS)

MR^4^: BCR-ABL1/ABL1 ratio of ≤ 0.01% on the IS

MR^4.5^: BCR-ABL1/ABL1 ratio of ≤ 0.0032% on the IS

^i^ Censored at time of relapse or death

**Table 2:** Treatment-Associated Toxicities

Variables ^a^	All Patients (*n* = 18)	TSR ^b^ (*n* = 9)	7 + 3 ^c^ (*n* = 9)
**Baseline Characteristics**			
Febrile neutropenia, n (%)	18 (100)	9 (100)	9 (100)
Infection, n (%)	8 (44.4)	5 (55.6)	3 (33.3)
Significant bleeding, n (%)	3 (16.7)	1 (11.1)	2 (22.2)
Severe organ dysfunction ^d^			
Median number [IQR]	1 [0–1]	1 [1–2]	1 [0–1]
Cardiac, n (%)	4 (22.2)	4 (44.4)	0 (0)
Pulmonary, n (%)	6 (33.3)	3 (33.3)	3 (33.3)
Gastrointestinal, n (%)	4 (22.2)	4 (44.4)	0 (0)
Renal, n (%)	3 (16.7)	1 (11.1)	2 (22.2)
Hepatic, n (%)	3 (16.7)	1 (11.1)	2 (22.2)

*Abbreviations* aHSCT, allogeneic hematopoietic stem cell transplantation; CML, chronic myeloid leukemia; CR, complete remission; CRi, complete remission with incomplete hematologic recovery; TSR, time-sequenced regimen

^a^ All variables reported as median [IQR] unless otherwise stated

^b^ Time-sequenced regimens consisting of: fludarabine plus high-dose cytarabine and idarubicin (FIA or FLAG-Ida), cladribine plus high-dose cytarabine and idarubicin (CLIA), and cladribine plus high-dose cytarabine and mitoxantrone (CLAG-M)

^c^ Intensive chemotherapy consisting of 7 days of standard-dose cytarabine and 3 days of idarubicin or daunorubicin

^d^ Defined as Grade ≥ 3 toxicity in accordance with the Common Terminology Criteria for Adverse Events version 5

### Toxicity outcomes

All patients experienced febrile neutropenia with induction therapy of whom 55.6% of TSR- and 33.3% of “7 + 3”-treated patients had positive infectious diseases findings. Twelve patients (66.7%) experienced at least one grade ≥ 3 non-hematologic, end-organ toxicity which involved the lungs (*n* = 6; 33%), cardiovascular system (*n* = 4; 22.2%), gastrointestinal tract (*n* = 4; 22.2%), kidneys (*n* = 3; 16.7%), and liver (*n* = 3; 16.7%). Among patients who received a TSR or 7 + 3 induction chemotherapy, 33.3% and 11.1%, respectively, experienced grade ≥ 3 multi-organ toxicity (Table 3). Stratified by baseline ECOG score, 33.3% (*n* = 2/6) of patients with an ECOG of 2 or 3 compared to 16.7% (*n* = 2/12) of patients with an ECOG of 0 or 1, experienced grade ≥ 3 multi-organ toxicity. Lastly, patients with a higher blast percentage (≥ 50% vs. < 50% bone marrow blasts) at baseline also experienced more grade ≥ 3 multi-organ toxicity (33.3% vs. 16.7%). Receipt of ponatinib versus dasatinib did not affect the number of patients who experienced at least one (66.7% vs. 66.7%) or two (16.7% vs. 25%) grade ≥ 3 end-organ toxicities. Causes of death included septic shock (*n* = 3) alone or concurrent with cardiopulmonary arrest (*n* = 5) and fungal infection (*n* = 5), and fulminant liver failure (*n* = 1). Ten patients (71.4%) died with active disease.

**Table 3:** Induction and salvage therapy responses

Patient No.	Age (years)	Treatment	Hematologic Response	CSCR ^a^	Best Molecular Response ^b^	aHSCT	Relapse	Salvage Therapy	Response
1	68	7 + 3 dasatinib 140 mg	Refractory	N/A	-	No	-	None	-
2	57	7 + 3 ponatinib 45 mg	CRi	Yes- Minor	MR^4.5^	Yes	No	-	-
3	52	7 + 3 dasatinib 140 mg	Refractory	No	-	No	-	None	-
4	38	7 + 3 dasatinib 140 mg	CR	No	MR^4.5^	Yes	No	-	-
5	51	7 + 3 ponatinib 15 mg	CRi	No	No MR	No	Yes	FIA ponatinib	Refractory
6	36	7 + 3 dasatinib 140 mg	CR	Yes- Minor	MMR	No	No	-	-
7	29	7 + 3 dasatinib 100 mg	Refractory	No	-	No	-	MEC dasatinib	CRi
8	45	7 + 3 dasatinib 140 mg	CRi	Yes- Minor	No MR	No	No	-	-
9	46	7 + 3 dasatinib 100 mg	Refractory	No	-	No	-	Decitabine venetoclax	Refractory
10	26	CLIA dasatinib 100 mg	CR	No	No MR	No	Yes	FIA ponatinib	CRi
11	39	FLAG-Ida ponatinib 45 mg	Refractory	No	-	No	-	Azacitidine venetoclax	Refractory
12	67	FLAG-Ida dasatinib 140 mg	Refractory	N/A	-	No	-	None	-
13	51	CLAG-M dasatinib 100 mg	Refractory	No	-	No	-	G-CLAM ponatinib 45 mg	Refractory
14	71	CLAG-M dasatinib 100 mg	CR	Yes- Major	MR^4.5^	Yes	No	-	-
15	72	CLAG-M dasatinib 70 mg	CR	No	MMR	Yes	No	-	-
16	53	FLAG-Ida ponatinib 45 mg	Refractory	No	-	No	-	None	-
17	39	CLIA ponatinib 30 mg	CRi	Yes- Major	No MR	No	Yes	None	-
18	56	FLAG-Ida ponatinib 30 mg	CRi	No	No MR	No	Yes	Azacitidine venetoclax	Refractory

*Abbreviations* 7 + 3, 7 days of low-dose cytarabine and 3 days of idarubicin or daunorubicin; aHSCT, allogeneic hematopoietic stem cell transplantation; CLIA, cladribine plus high-dose cytarabine and idarubicin; CLAG-M, cladribine plus high-dose cytarabine and idarubicin with granulocyte-colony stimulating factor priming; CR, complete remission; CRi, complete remission with incomplete hematologic recovery; CSCR, clinically significant cytogenetic response; FIA, fludarabine plus high-dose cytarabine and idarubicin; FLAG-Ida, fludarabine plus high-dose cytarabine and idarubicin with granulocyte-colony stimulating factor priming; IS, international scale; MEC, mitoxantrone, etoposide, high-dose cytarabine; MLFS, morphologic leukemia-free state; N/A, not available; NoMR, no molecular response

^a^ Defined as achieving at least a minor cytogenetic response (≤ 65%) after induction therapy. Minor and major response defined by presence of 0-35% and 36-65% of metaphases exhibiting the Ph(+) chromosome, respectively

^b^ MMR(MR^3^): BCR-ABL1/ABL1 ratio of ≤ 0.1% on the international scale (IS)

MR^4^: BCR-ABL1/ABL1 ratio of ≤ 0.01% on the IS

MR^4.5^: BCR-ABL1/ABL1 ratio of ≤ 0.0032% on the IS

### Overall and event-free survival

**Fig. 1:**
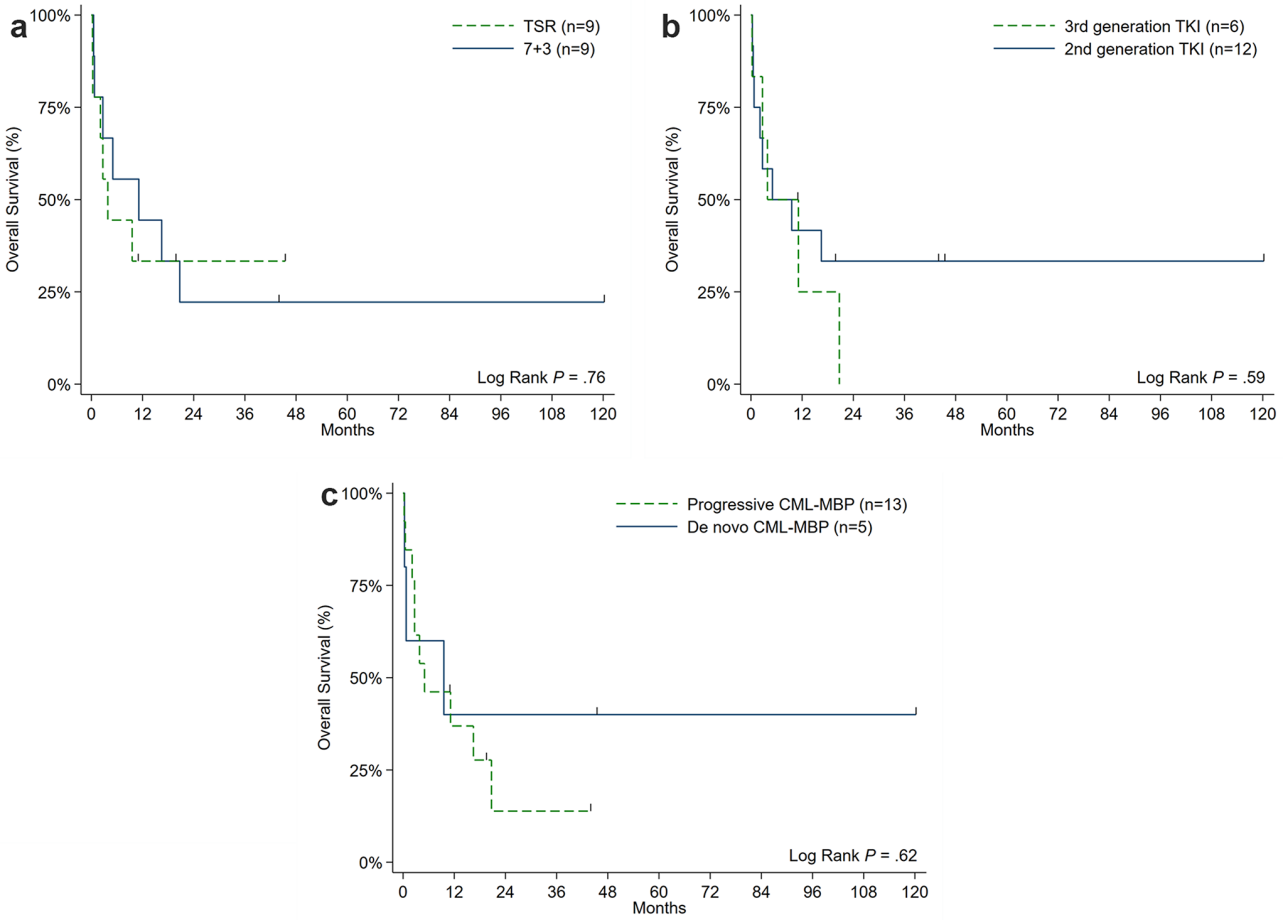
Overall survival of CML-MBP patients stratified by those who **a**) received TSR vs. 7 + 3 induction chemotherapy, **b**) received 3rd versus 2nd generation TKI therapy, and c) progressed from chronic or accelerated phase CML or presented with de novo CML-MBP. *Abbreviations* CML, chronic myeloid leukemia; MBP, myeloid blast phase; tyrosine kinase inhibitor; TSR, time-sequenced regimen

**Fig. 2:**
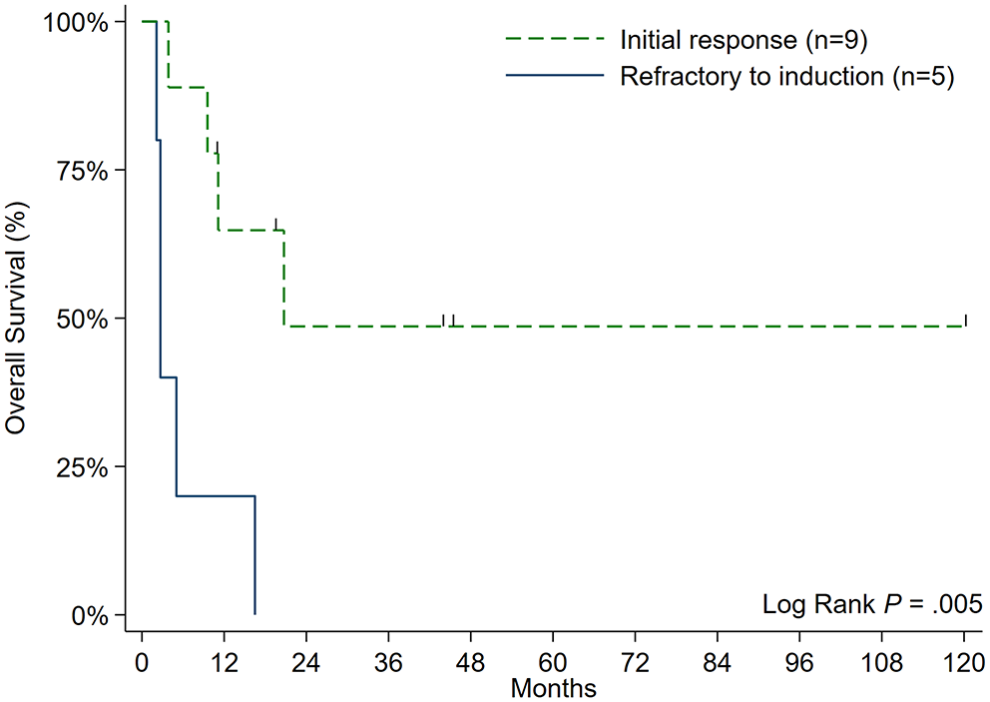
Overall survival of CML-MBP patients stratified by those who responded or did not respond to induction therapy. Abbreviations: CML, chronic myeloid leukemia; MBP, myeloid blast phase

**Fig. 3:**
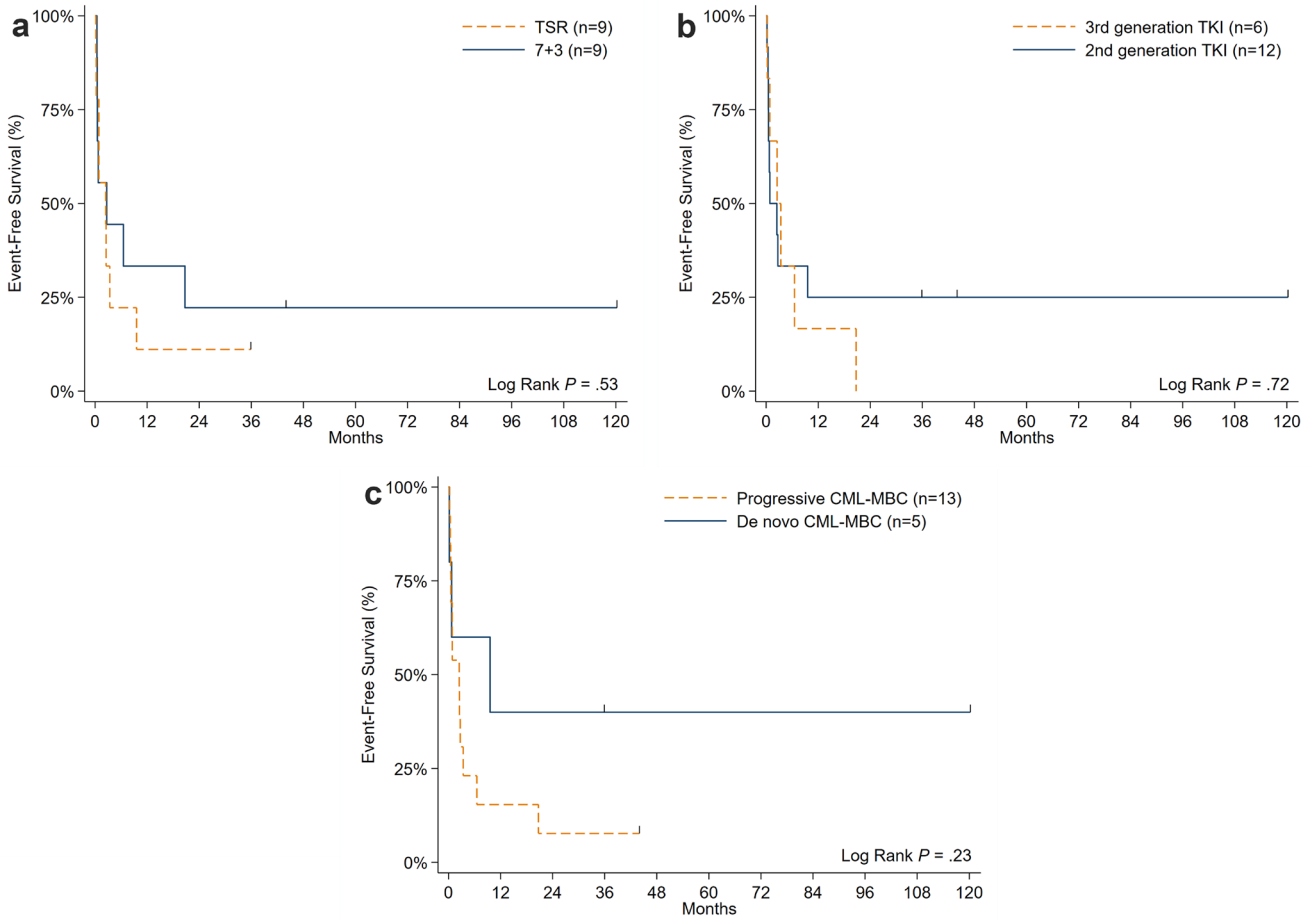
Event-free survival of CML-MBP patients stratified by those who **a**) received TSR vs. 7 + 3 induction chemotherapy, **b**) received 3rd versus 2nd generation TKI therapy, and **c**) progressed from chronic or accelerated phase CML or presented with de novo CML-MBP. Abbreviations: CML, chronic myeloid leukemia; MBP, myeloid blast phase; TKI, tyrosine kinase inhibitor; TSR, time-sequenced regimen

With a median follow-up of 44.5 months among survivors, OS was not significantly different between patients who received a TSR vs. 7 + 3 chemotherapy (31.8% vs. 44.4% at 1-year; *P* = 0.76), ponatinib vs. dasatinib (30% vs. 41.7% at 1-year; *P* = 0.59), or patients who presented with progressed vs. *de novo* CML-MBP (37.8% vs. 40% at 1-year; *P* = 0.62) (Fig. 1a and c). OS did not differ between patients who proceeded (*n* = 4) or did not proceed to aHSCT (75% vs. 55.6% at 1-year; *P* = 0.78); however, both cases of mortality in the aHSCT cohort were attributed to non-relapse causes. The only significant variable associated with higher OS was initial attainment of CR/CRi after induction therapy (65.2% vs. 20% at 1-year; *P* = 0.005) (Fig. 2), stressing the impact of optimizing upfront CML-MBP therapy. Median EFS was 2.5 months for the whole cohort. EFS was not significantly different between patients who received a TSR vs. “7 + 3” chemotherapy (11.1% vs. 33.3% at 1-year; *P* = 0.53), ponatinib vs. dasatinib (16.7% vs. 25% at 1-year; *P* = 0.72), or patients who presented with progressed vs. *de novo* CML-MBP (16.7% vs. 25% at 1-year; *P* = 0.23) (Fig. 3a and c**)**.

## Discussion

Treatment outcomes for patients with CML who progressed to myeloid blast phase remain dismal, with studies reporting median OS at 12 months or less [9]. Targeted therapy with a TKI remains a key component of treatment for CML-MBP. Although in this study we were able to analyze a relatively small number of patients, we found no benefit from modifications in the chemotherapy component, particularly for those that received an intensified regimen. Due to its rarity, treatment selection is often guided by small retrospective studies or case reports. The recently published MATCHPOINT study was a multicenter, prospective phase 1/2 trial that sought to optimize the efficacy and tolerability of the TSR, FLAG-IDA, with ponatinib [10]. Of 9 treated CML-MBP patients, hematologic response was achieved in only one (14.3%) of 7 evaluable patients, with two experiencing early treatment-related mortality. A more promising approach to treatment is the combination of a TKI with therapies that possess more selective targeting than those offered by traditional chemotherapy.

With an increased understanding of the molecular mechanisms that drive CML-MBP transformation, including hypermethylation of several significant oncogenes, an emphasis on epigenetic reprogramming may be associated with improved outcomes compared to more intensive chemotherapy. Two epigenetic modifying therapies, decitabine and azacitidine, have demonstrated high hematologic response rates of 44.4% (*n* = 8/18) and 71.4% (*n* = 5/7), respectively, in conjunction with TKI therapy [11, 12]. In the analysis performed by Saxena, et al., CR/CRi was similarly achieved in 55% (*n* = 11/20) of patients receiving decitabine in combination with a TKI, although 35% received the first generation TKI, imatinib.^7^ When patients receiving imatinib were excluded, CR/CRi increased to 76.9% (*n* = 10/13).

The introduction of the BCL-2 inhibitor, venetoclax, has brought about significant changes in the AML treatment landscape. With overexpression of BCL-2 in CML-MBP and preclinical data showing synergy of venetoclax and BCR-ABL targeting TKIs in eradication of leukemic stem cells, this potent combination could improve response rates for CML-MPB patients. In a single-center, retrospective cohort study, eight evaluable CML-MBC patients received venetoclax and TKI-based combinational therapy (4 with intensive chemotherapy and 4 with decitabine) [13]. An impressive 75% objective response was reported (5 patients with CR/CRi and 1 with a partial response) of whom 50% of patients had relapsed/refractory CML-MBP. All patients were previously treated with TKI therapy, and received a median of 4 (range, 2–8) prior lines of therapy. Interestingly, all 4 patients who received decitabine, venetoclax, and a BCR-ABL targeting TKI achieved a hematologic response with 75% demonstrating minimal residual disease negativity by flow cytometry. Acknowledging the fundamental precautions of cross-comparing data across studies and the relatively small number of patients in these studies, the combination of venetoclax, a BCR-ABL targeting TKI, and decitabine may prove highly effective in achieving hematologic response and bridging patients to aHSCT in transplant eligible candidates. A phase 2 study investigating this combination is currently underway (NCT04188405) [14]. The combination of venetoclax, TKI, and intensive chemotherapy may be considered as a reasonable alternative in young, fit patients; however, dose attenuations (i.e., reduction of treatment days from 5 to 3) are likely warranted given the lack of improvement seen with chemotherapy intensification. Although more grade ≥ 3 multi-organ toxicity was noted with chemotherapy intensification, a higher number of TSR-treated patients had ECOG scores of 2 or 3, which may have influenced the toxicity rates seen in our cohort. Patients with higher disease burden based on presenting bone marrow blasts also exhibited more frequent grade ≥ 3 multi-organ toxicity however, a higher percentage of 7 + 3-treated patients had blasts ≥ 50% (Table 2).

This study has several limitations, including a single-center, retrospective design with a small sample size. Our ability to make definitive conclusions about causality and efficacy are constrained as a result and our analysis should be considered exploratory, requiring confirmation in a larger cohort. In contrast to the results presented in this analysis however, many previous studies investigated intensive chemotherapy with imatinib, which has since been deemed inferior to second or third generation TKIs [2, 3, 15]. This highlights the need for additional data, with second or third generation TKIs.

In conclusion, we suggest that intensification of upfront chemotherapy combined with ponatinib does not seem to improve treatment outcomes in CML-MBP patients. With emerging data supporting the utilization of hypomethylating agents or chemotherapy with BCL-2 inhibitors, future studies focused on combining these therapies with second or third generation TKIs are warranted.

## Data Availability

The data that support the findings of this study are available from the corresponding author upon reasonable request.
